# Does Foveal Hypoplasia Affect Emmetropization in Patients with Albinism?

**DOI:** 10.3390/children10121910

**Published:** 2023-12-11

**Authors:** Line Kessel, Christine Dahlgren Bohnsack Kjølholm, Joaquim Torner Jordana

**Affiliations:** 1 Department of Ophthalmology, Copenhagen University Hospital—Rigshospitalet, 2100 Copenhagen, Denmark; christine.kjoelholm@regionh.dk (C.D.B.K.); joaquim.torner.jordana@regionh.dk (J.T.J.); 2 Department of Clinical Medicine, University of Copenhagen, 2200 Copenhagen, Denmark

**Keywords:** albinism, emmetropization, foveal hypoplasia, refractive errors, refractive development

## Abstract

(1) Background: The aim of the study was to describe refractive development from early childhood to adulthood in Danish patients with albinism and to evaluate the effect of foveal developmental stage on refractive development; (2) Methods: Patients with a clinical diagnosis of ocular or oculocutaneous albinism were invited for a refractive evaluation and comprehensive phenotyping including macular optical coherence tomography (OCT) scans. Foveal hypoplasia was graded based on OCT from 0 (normal) to 4 (absence of any signs of foveal specialization). Medical files were reviewed for historical refractive values in individual patients; (3) Results: Hyperopia (spherical equivalent refraction (SEQ) of ≥+1 Diopter (D)) was common in both children (81.3%) and adults (67.1%). The lower prevalence of hyperopia in adults was predominantly explained by increasing astigmatism with age. Emmetropization (>2D change from before 3 years to adolescence) was seen in 22.2%. There was no influence on foveal hypoplasia grade on the degree of refractive errors throughout life; (4) Conclusions: We found that emmetropization was uncommon in Danish patients with albinism and that the degree of foveal developmental stage did not influence emmetropization or the distribution of refractive errors. High degrees of hyperopia and astigmatism were common. These results indicate that fear of impeding emmetropization should not refrain the clinician from providing adequate correction for refractive errors in young children with albinism.

## 1. Introduction

Albinism is a rare genetic disease where the most classic phenotype is hypopigmentation of all ocular tissues from eye lashes to the retina. In addition to the hypopigmentation, patients have foveal maldevelopment, abnormal decussation of fibers in the optical tract and alterations in the visual cortex [1]. Patients with albinism have a fair complexion with hypopigmentation or no pigmentation of hair and skin. The hypopigmentation of skin increases the risk of skin cancers [2] especially in countries with high levels of ultraviolet radiation. Skin cancers are an important cause of increased mortality in African patients with albinism [3].

Most affected patients have nystagmus and poor visual acuity. The poor vision is partly thought to be caused by foveal hypoplasia and partly by the abnormal central visual pathways. Albinism is a common cause of low vision in childhood [4] and a frequent cause of congenital nystagmus [5]. Nystagmus may in itself reduce visual acuity but often there is a causative relationship between the nystagmus and the low visual acuity, e.g., foveal hypoplasia in the case of patients with albinism or poor retinal function in the case of children with retinal diseases such as Lebers congenital amaurosis or achromatopsia.

Refractive errors are common in patients with albinism and may further add to visual impairment in case they are not corrected by proper optical prescription due to the blurred and unfocused image on the retina. High degrees of astigmatism, hyperopia, and myopia are frequently reported [6,7]. Especially astigmatism is associated with a high risk of amblyopia development for low grades of refractive errors whereas hyperopia and myopia can also be associated with risk of amblyopia but will usually require higher degrees of refractive error. Amblyopia may lead to life-long visual loss if not treated in early childhood.

Most healthy children are hyperopic in early infancy but as the eye grows, the refractive powers of the eye change towards emmetropia to match the geometrical status of the eye [8]. This process is called emmetropization. It is not fully understood what drives emmetropization in humans but visual cues are required [9]. Cortical processing of visual inputs is, however, not required to drive emmetropization and it has been suggested that one or several humoral factors from the retina or retinal pigment epithelium regulates scleral growth directly [10]. Based on studies on monkeys [11] where the fovea was ablated at 3 weeks of age, the fovea is not expected to play a role in emmetropization or the risk of developing form deprivation myopia. There is, however, little evidence from human studies as to the role of the fovea in regulation of normal eye growth. 

Patients with albinism have reportedly an impaired emmetropization and it has been suggested that this is related to albinism rather than foveal hypoplasia [12]. These findings have been questioned due to the differences in degrees of foveal hypoplasia in the patients with and without albinism in that particular study [13]. Animal studies have also found an impaired emmetropization in albino chick [14]. Form deprivation myopia seems to be a greater risk in albino guinea pig [15]. 

The aim of the study was to evaluate refractive development and distribution of refractive errors in Danish patients with albinism and to evaluate the role of foveal maturation on these parameters. 

## 2. Materials and Methods

Patients with a clinical diagnosis of ocular or oculocutaneous albinism who had previously been seen at the Eye Clinic at Kennedy Center, Rigshospitalet, Denmark, were invited by mail to participate in the study. The Eye Clinic at Kennedy Center is a national, tertiary referral center for patients (children and adults) with low vision mainly due to genetic disorders. The study included phenotypic and genotypic characterization as well as an offer of optical rehabilitation. Details of the study procedures, genotype-phenotype correlations, and results of optical rehabilitation have been published previously [16,17].

In the present study we evaluated refractive development using historical information available in the patients’ medical files. All included patients had been seen at least once previously at the clinic. Most patients had been followed at several time points throughout their entire life at the clinic starting in early childhood and continuing into adulthood. The longest available follow-up was nearly 70 years. Many different methods had been used throughout the years to obtain information on the refractive status of the patients. In older medical files it was not always possible to discern which method had been used. Whenever possible we noted the cycloplegic refraction based on objective measurements (e.g., retinoscopy or autorefraction). 

In addition to the historical data, the patients included in this report underwent an updated examination by an optometrist specialized in low vision and an ophthalmologist. The refractive status of patients was assessed by objective (retinoscopy and autorefraction using Retinomax K-plus, Righton, Japan) and subjective refraction. For pre-presbyopic patients, refractive evaluation after cycloplegia using cyclopentolate 1% was performed and used in the analyses below.

The best corrected distance of visual acuity was measured using the ETDRS chart (Precision Vision, Woodstock, IL, USA) at 4- or 1-m distance depending on the visual acuity of the patient. Visual acuity is reported as the number of letters read. A score of 85 letters corresponds to 20/20 (or logMAR 0.0). A score of 70 letters correspond to 20/20 (or logMAR 0.3). A score of 50 letters correspond to 20/100 (or logMAR 0.7) and a score of 35 letters correspond to 20/200 (or logMAR 1.0).

Digital slit lamp photographic images and films of the anterior segment were recorded to determine the presence and direction of nystagmus.

**Figure 1:**
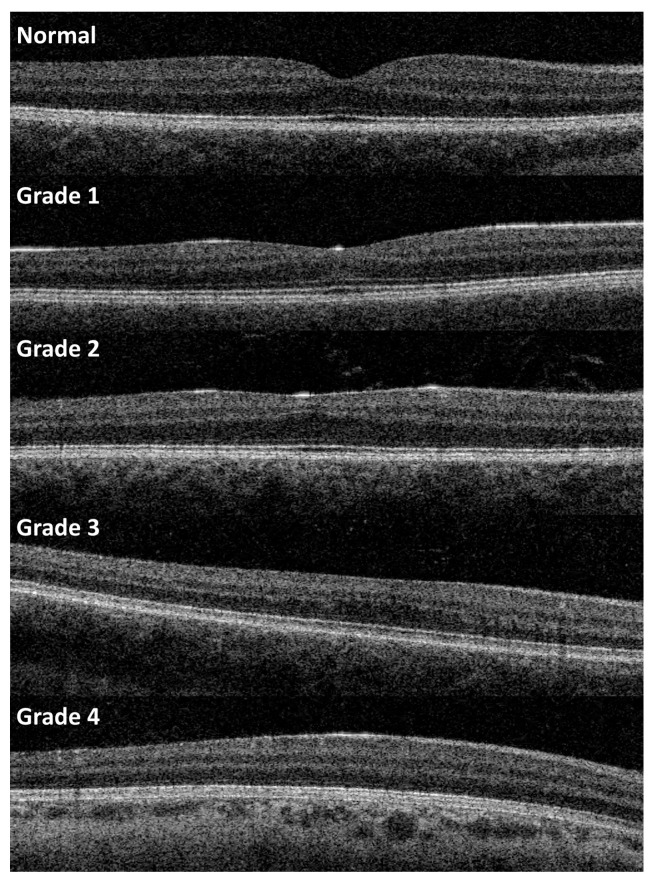
The figure shows OCT scans of patients with different grades of foveal hypoplasia according to the grading suggested by Thomas et al. [18]. Grade 0 indicates a normal fovea. Grade 1 is characterized by absence of extrusion of plexiform layers, grade 2 by absence of a foveal pit, grade 3 by absence of lengthening of the outer segments, and grade 4 by absence of widening of the outer nuclear layer.

Foveal developmental stage was evaluated according to Thomas et al. [18] using 3D macular scans (6.0 mm × 6.0 mm, 512 × 128) centered on the central macula (Topcon OCT, OCT-2000, Topcon, Japan), see Figure 1. If 3D scans could not be obtained, for example due to severe nystagmus, we used a line scan centered on the central macula. 

Data were presented using descriptive statistics. Associations between categorical and continuous data were analyzed using ANOVA. A *p*-value of 0.05 was considered statistically significant. There was a high degree of correlation of refractive errors between the two eyes (Pearson’s correlation coefficient 0.77 (*p*-value 0.4 for paired *t*-test) and 0.78 (*p*-value 0.6 for paired *t*-test) for subjective and objective measurements at the re-evaluation visit, respectively). Only data from the right eye are presented. Spherical equivalent refraction (SEQ) was calculated as the spherical error plus half the negative cylinder. Hyperopia was defined as SEQ ≥ +1.0 Diopter, myopia was defined as SEQ ≤ −1.0 Diopter, emmetropia was defined as SEQ between −1.0 and +1.0 Diopter.

## 3. Results

We included 96 patients with albinism, 46 (48%) were women and 50 (52%) were males. Twenty-nine (30%) patients had albinism caused by variants in *TYR* gene, 23 (24%) had *OCA2*, 14 (15%) had x-linked albinism with variants in *GPR143* gene, 13 (13%) had other genetically verified types of albinism and in the remaining 17 (18%) the genetic cause of albinism could not be established. Patients had been followed for a mean of 23.9 years (SD 14.2). The longest follow-up was 68.3 years. Follow-up was <1 year in 10 patients. The mean age at latest examination was 34.1 years (standard deviation (SD) 18.5). Nystagmus was prevalent with 67 (68%) having horizontal nystagmus, 9 (9%) had rotatory nystagmus, 5 (5%) had mixed nystagmus, and 6 (6%) did not have nystagmus. In nine patients, videos demonstrating ocular movements were unavailable. Visual acuity was 54.6 ETDRS letters (mean, SD 13.0) for all patients, and 64.1 (15.0) for those with foveal hypoplasia grade 0–2, 60.8 (11.6) for those with grade 3, and 50.8 (11.9) for those with grade 4. 

**Figure 2:**
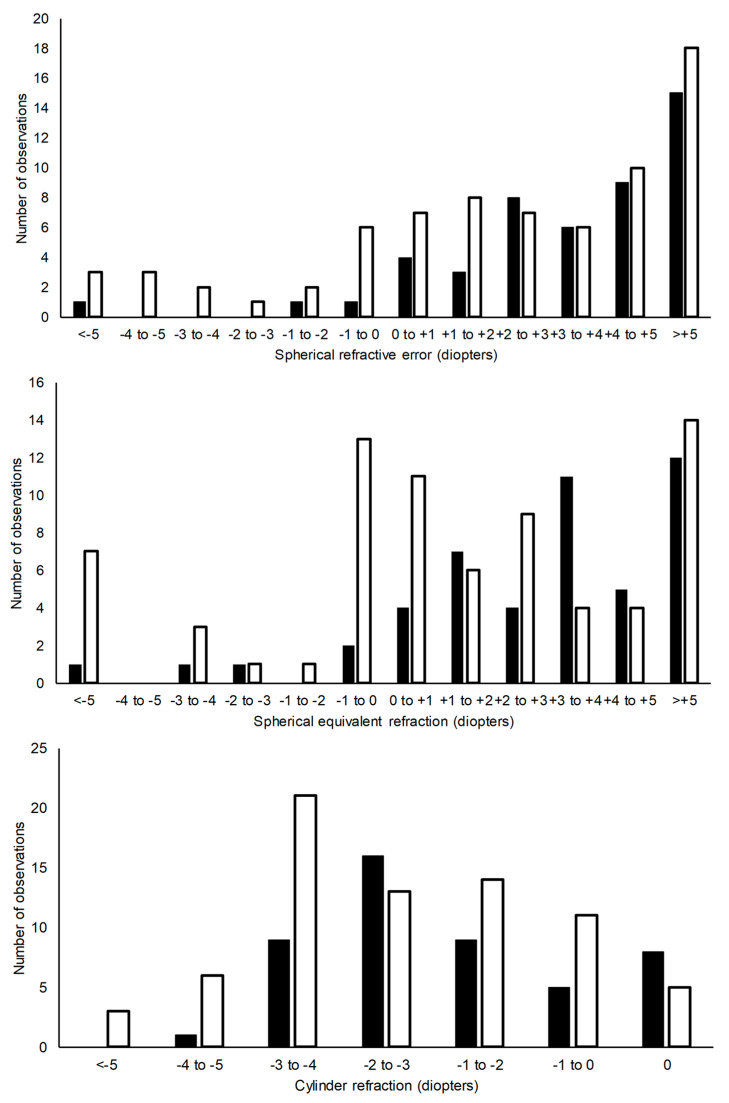
The figure shows the distribution of refractive errors in children ≤5 years of age (black bars) and adults ≥18 years of age (white bars). Each patient is only represented once in childhood and as adult but may appear in both age groups. The upper diagram shows spherical refractive error (SER), middle diagram shows spherical equivalent refraction (SEQ), and lower diagram shows astigmatic refractive errors.

Most patients had high degrees of refractive errors. In preschool children (≤5 years of age), 81.3% had a hyperopic spherical equivalent refraction (SEQ) of ≥+1 Diopter versus 67.1% in adults (≥18 years of age) whereas only 6.3% had a myopic SEQ in childhood versus 16.4% in adults. The change towards myopia in adulthood was only partly explained by decreased spherical refraction, and to a greater extent by increasing astigmatism as shown in Figure 2.

**Figure 3:**
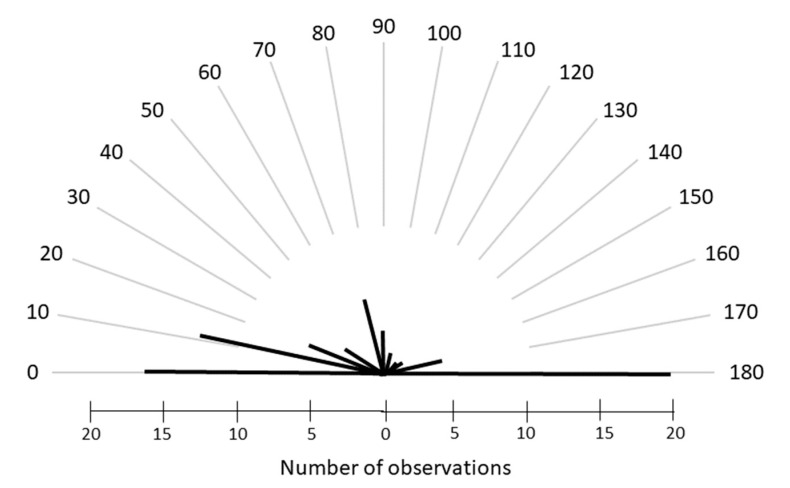
The graph shows the distribution of astigmatism axis (negative cylinder) for adults >18 years.

Few patients did not have astigmatism. The majority of patients had an astigmatism with a negative cylinder axis centered horizontally and few had astigmatism with a vertical axis, see Figure 3. A total of 78.3% had astigmatism with the negative cylinder at the horizontal axis +/− 10 degrees.

There were no significant differences in the degree of spherical or cylinder refractive errors or spherical equivalent refraction between the different degrees of foveal hypoplasia in children (≤5 years or age) or adults (≥18 years of age) (ANOVA, all *p*-values > 0.2), see also Table 1. 

**Figure 4:**
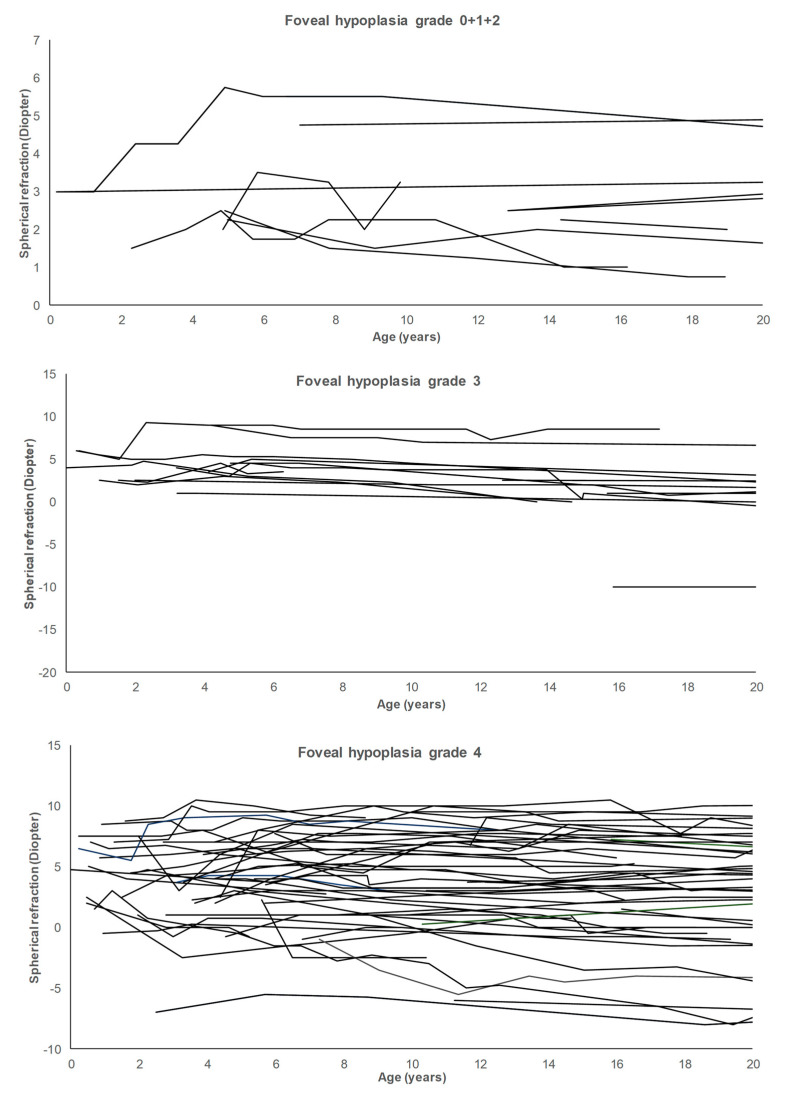
The spaghetti plot demonstrates development of spherical refraction in individual patients with albinism during childhood. The three plots show development for patients with foveal hypoplasia grade 0 + 1 + 2 (upper graph), grade 3 (middle graph), and grade 4 (lower graph). Only patients with multiple observations are shown and observations for each patient are connected by lines.

To evaluate emmetropization on an individual level, we compared refractive errors in patients with observations ≤3 years of age and between 10 and 15 years of age (n = 33). In 77.8%, the spherical refractive error had decreased ≤1 diopter, the decrease was 1–2 diopters in 3.7% and > 2 diopters in 18.5%. For spherical equivalent refraction, the decrease from ≤3 years of age to 10–15 years of age was ≤1 diopter in 63.0%, it was 1–2 diopters in 14.8% and it was >2 diopters in 22.2%. Spaghetti plots showing changes in refractive errors for individual patients during the follow-up period are shown in Figure 4. 

## 4. Discussion

We evaluated refractive errors and emmetropization in Danish patients with albinism. We found that refractive errors, especially hypermetropia and astigmatism, were common and that few patients showed signs of emmetropization and even fewer developed myopia or had myopia at the time of first examination. We did not find that the degree of foveal hypoplasia influenced the distribution of refractive errors or emmetropization, but it should be kept in mind that most patients had grade 3 or 4 foveal hypoplasia and very few had milder degrees. The effect of foveal hypoplasia on spherical equivalent refractive error was previously evaluated in a smaller study on 33 patients with albinism [12]. This study also showed widely overlapping confidence intervals for SEQ in patients with albinism although only patients with foveal hypoplasia grade 3 and 4 were included.

We found that degree of astigmatism increased with age and that this led to a decrease in spherical equivalent refraction. Others have found a high prevalence of astigmatism and increase in the degree of astigmatism in children with idiopathic congenital nystagmus [19]. The prevalence and degree of astigmatism is reportedly even greater in children with albinism than in other types of congenital nystagmus [20]. An increase in astigmatism during childhood in patients with albinism has been reported by others [21]. It has been suggested that the astigmatism may be caused by mechanical remodeling of the corneal surface as the eye moves between the eye lids. This theory is supported by our study where most patients had with-the-rule astigmatism and they also mainly had horizontal nystagmus. We did, however, find that the degree of astigmatism was greatest in adults with non-horizontal nystagmus. We did not have enough patients with non-horizontal nystagmus and refractive information available in both childhood and as adults to evaluate whether the direction of nystagmus influenced the development of astigmatism over time.

Understanding the process of emmetropization is important with the global trend in increasing myopia prevalence. Many countries have reported dramatic changes in myopia prevalence in recent years [22]. It has been projected that 50% of the world population will be affected by myopia in 2050 and that 10% of the world population will have high myopia [23] that is associated with an increased risk visual loss. Each diopter of myopia is associated with an increased risk of visual loss and nearly all patients with high myopia will experience visual loss late in life [24]. These serious consequences of myopia drive the current research focus on what drives myopia development. In most patients, myopia seems to be a result of a failed emmetropization process.

Most of our current knowledge on emmetropization is derived from animal studies. They have shown that post-natal emmetropization is an intricate and local process where eye growth is regulated by visual inputs that do not require post-ocular signal processing and that form deprivation will lead to myopia [25]. Inducing hyperopic defocus in chicks will increase eye growth and myopic defocus will reduce eye growth [26]. As shown in this study, this regulatory process does not seem to come into play in patients with albinism as even those with very high degrees of hyperopia did not seem to reduce the amount of spherical refraction during childhood. Healthy children are expected to undergo significant refractive development in the first years of life. Most children are born mildly hyperopic, usually <3–4 Diopters, and will reach emmetropia around 7 to 9 years of age [8,27,28]. 

High myopia was found in very few patients in this study. The myopic patients in our study all had foveal hypoplasia grade 3 or 4 except for two who did not have OCT scans available. A nearly equal distribution of myopia and hyperopia in patients with albinism was found in a large Indian study even in children [29]. A small study from the USA found an overrepresentation of hyperopia in patients with albinism [6]. There is no obvious explanation why there is a difference in the distribution of refractive errors between different countries. 

In our study, visual acuity was low in myopic patients (52 to 36 ETDRS letters) suggesting that form deprivation may potentially play a role in myopia in patients with albinism. As shown in this study and by others [30] there is a correlation between poor visual acuity and foveal hypoplasia grade.

Ambient light levels and the chromatic composition of ambient light has been demonstrated to influence eye growth in several animal models [31] and time spent outdoor is a well-established protecting factor against myopia development in children [32]. In albinism, an increased amount of light is expected to reach the retina and choroid due to the lack of pigmentation in the iris and retinal pigment epithelium. It is, however, at this stage highly hypothetical that the increased amount of light should influence the lack of emmetropization observed in patients with albinism. It should also be kept in mind that many patients with albinism will use some form of filter, colored or neutral, to reduce photosensitivity. The role of spectral composition on eye growth in humans is unknown. Patients with achromatopsia, and no cone function able to detect the different chromatic stimuli, have a near-normal emmetropization [33]. 

One of the aims of this study was to evaluate the role of foveal developmental stage on emmetropization. Animal studies have shown that ablation of the fovea does not impair emmetropization [11]. In our study, we did not find evidence of normal emmetropization in patients with albinism no matter their foveal developmental stage.

We found that most patients had grade 3 or 4 foveal hypoplasia. Others have reported that foveal hypoplasia grade 1–4 are almost equally common in patients with albinism [34] whereas others have found grade 4 to be more common in albinism and especially in ocular albinism [35]. However, we had very few patients with normal or mild degrees of foveal hypoplasia in our study. To evaluate the direct effect of foveal hypoplasia on emmetropization in humans, different cohorts with a greater proportion of milder degrees of foveal hypoplasia, e.g., *FRMD7*-related foveal hypoplasia [35] may need to be studied.

We included 96 patients and based on the birth prevalence of albinism in Denmark [36] and a population of ~6 million, we included nearly ¼ of all Danish patients with albinism. Thus, although our study could potentially be biased towards inclusion of more severe cases, we believe that the distribution of foveal hypoplasia grades was representative for Danish patients with albinism. The role of foveal developmental stage on emmetropization in humans remains unclear and further studies on other populations with different causes for foveal hypoplasia are welcomed.

Albinism is associated with poor visual acuity due to incomplete foveal development and possibly also other factors, such as iris translucency and optic nerve abnormalities with misrouting of the optic tract. At the same time, albinism is characterized by high refractive errors and it can be debated when and to which extent the refractive errors should be corrected in babies as some might fear that providing full correction at the initial visit may impair emmetropization. The American Academy of Ophthalmology recommends correcting refractive errors over 6 Diopters of hyperopia or 5 Diopters of myopia in infants <1 year [37], but does not provide specific recommendations for infants with albinism. However, as our data show, there is little evidence to suggest that patients with albinism have normal emmetropization. Others have argued that one should not hold back prescribing for hyperopia in childhood to improve visual function due to the fear of impacting emmetropization [38]. A study on patients with albinism indicated that visual function was better if glasses were started early in life [39]. Thus, it seems advisable to prescribe the amount of correction believed to improve visual function even in small babies with albinism. 

Our study has some limitations. Most importantly, we relied on retrospective data collected over many years in a non-uniform way by different health care professionals using the examination modalities that had been available to them at the time the examinations were performed. A well-designed prospective study would have been better from a methodological standpoint but albinism is a rare disease. The Danish birth prevalence of albinism is reported to be 1 in 14,000 live births [36] and with an annual number of births of around 60,000 in Denmark it would take >20 years to collect the same number of individuals as in our study in a prospective study if every new-born with albinism was included. This is unrealistic. Thus, for the present purpose—to evaluate refractive development in patients with albinism—the present study design was the only realistic option. A second limitation is that non-uniform methods of refraction had been used and the method used was not always easy to discern from the medical file. However, all examinations had been performed at the same clinic that throughout the entire period was a tertiary referral center with specially trained low vision specialists including optometrist and ophthalmologists who have trained newer generations of clinicians and with a low turn-over of staff. Thus, methods of refractive evaluation are believed to be robust over time. Finally, we included patients who had been given a clinical diagnosis of albinism. In the majority of patients, the clinical diagnosis was confirmed by genetic work-up but in a few patients, genetic work-up failed to support the clinical diagnosis. However, when we applied diagnostic criteria suggested by others [34], patients (or also affected family members) fulfilled those diagnostic criteria [17].

## 5. Conclusions

In conclusion, we found that emmetropization was disturbed in patients with albinism and that the distribution and development of refractive errors was not influenced by foveal developmental stage.

## Figures and Tables

**Table 1:** Refractive errors, foveal hypoplasia, and age of patients.

	0–2 Years	3–5 Years	6–8 Years	9–12 Years	13–17 Years	≥18 Years
Spherical refraction (SER)						
Grade 0–2	2.6 (1.5) [3]	3.6 (1.3) [7]	4.0 (1.5) [8]	3.1 (149) [10]	1.0 (4.4) [12]	21.0(4.0) [18]
Grade 3	3.8 (4.0) [6]	4.4 (2.9) [9]	5.1 (2.2) [7]	3.8 (2.7) [9]	2.5 (3.0 [7]	1.7 (1.7) [13]
Grade 4	4.6 (3.9) [21]	4.1 (3.7) [37]	4.2 (3.8) [38]	3.7 (4.0) [40]	3.9 (4.2) [35]	3.5 (4.6) [45]
Cylinder refractive error						
Grade 0–2	−1.7 (1.4) [3]	−2.2 (1.2) [7]	−2.2 (1.0) [8]	−2.7 (1.5) [10]	−2.7 (1.5) [12]	−2.7 (1.7) [18]
Grade 3	−1.7 (1.8) [6]	−1.4 (1.5) [9]	−2.0 (1.4) [7]	−3.0 (2.0) [9]	−2.4 (1.6) [7]	−2.4 (1.9) [13]
Grade 4	−2.0 (0.8) [21]	−2.1 (1.2) [37]	−2.4 (1.1) [38]	−2.2 (1.6) [40]	−2.7 (1.2) [35]	−2.6 (1.3) [45]
Spherical equivalent refractive error (SEQ)						
Grade 0–2	1.8 (1.4) [3]	2.6 (1.3) [7]	2.9(1.4) [8]	1.8 (1.6) [10]	1.8 (1.6) [12]	−0.3 (3.8) [18]
Grade 3	3.0 (3.9) [6]	3.7 (1.7) [9]	4.0 (1.8) [7]	2.3 (2.7) [9]	1.3 (3.1) [7]	0.5 (1.2) [13]
Grade 4	3.6 (3.9) [21]	3.0 (3.8) [37]	3.05 (4.0) [38]	2.6 (41) [40]	2.6 (4.2) [35]	3.9 (3.7) [45]

Foveal hypoplasia was graded according to Thomas et al. [18]. Numbers are presented in mean (standard deviation) [number of patients]. Refractive errors are presented in Diopters. Each patient is only represented once for each age group but may be represented in several age groups. Foveal hypoplasia grade was not available in 12 participants.

## Data Availability

The data presented in this study are available upon reasonable request and given data sharing and collaboration agreements from the corresponding author. The data are not publicly available due to concerns for patient privacy given the rarity of the condition studied.
